# A new subspecies of Peucedanum officinale L. subsp. album (Apiaceae) from the eastern part of the Iberian Peninsula

**DOI:** 10.3897/phytokeys.131.32173

**Published:** 2019-09-02

**Authors:** Javier Martínez-Fort, Maela León, Maria P. Donat-Torres

**Affiliations:** 1 Col. Departamento Ecosistemas Agroforestales, C/Constitució 5, 46727 Real de Gandia, Spain Col. Departamento Ecosistemas Agroforestales Real de Gandia Spain; 2 Instituto Agroforestal Mediterráneo, Universitat Politècnica de València, Camino de Vera s/n, 46022Valencia, Spain Universitat Politècnica de València Valencia Spain; 3 Instituto de Investigación para la Gestión Integrada de Zonas Costeras (IGIC), Universitat Politècnica de València, Carretera Nazaret-Oliva s/n, 46730 Gandia, Spain Universitat Politècnica de València Gandia Spain

**Keywords:** Umbelliferae, Spain, taxonomy, subsp. nov., thermomediterranean, nrITS

## Abstract

We describe Peucedanum officinale L. subsp. album Martínez-Fort & Donat-Torres **subsp. nov.**, in which we grouped the thermomediterranean populations scattered along the eastern part of the Iberian Peninsula. The characters that differentiate this new subspecies from other infraspecific taxa in *Peucedanum officinale* are its canaliculated leaflet, the inflorescences much branched and lack of dominant terminal umbels, the umbels are few rayed, sometimes sessile and lateral, the petals are white and the fruit pedicels short, the same or shorter in length than the fruit. We provide here a full description of the new subspecies based on herbarium specimens and field measurements, as well as providing dichotomous keys to the subspecies within *P. officinale*. In addition, we provide a comparison of the ITS sequences of nrDNA with the most closely related taxons.

## Introduction

*Peucedanum* L. 1753 is one of the most complex genera in the Apiaceae family. Based on the morphological characteristics of the fruit, according to the traditional classification systems, it is characterised by a strong dorsal compression and winged side ribs as in Drude (1898). It is included within the Peucedaneae tribe, subtribe Ferulinae and has been defined as *Peucedanum* sensu amplissimo. In addition, this genus is broadly represented by 29 species in European flora (Tutin 1968), of which 10 are present in Iberian flora (Guillén and Laínz 2003). In the grouping of Pimenov and Leonov, *Peucedanum* sensu lato includes 100–120 scattered species throughout the Old World and the need for its reduction into natural groups has been proposed (Pimenov and Leonov 1993, Downie et al. 2000).

On the other hand, phylogenetic studies, based on the ITS rDNA sequences, define “*Peucedanum* sensu stricto clade including taxa that are very similar with respect to their ITS sequences and they are very related in habit, sharing not only fruit characters but also vegetative features, like linear-ﬁliform leaf lobes” and it has been regrouped in the Tribe Selineae Spreng (Spalik et al. 2004). Molecular phylogenetic studies confirm the separate taxonomic status of the other taxa which have been accepted as separate genera (Spalik et al. 2004, Winter et al. 2008, Downie et al. 2010). Therefore, the genus is now reduced to a few species and the type of the genus is *Peucedanum officinale* L.

The morphological differences between some populations from the central and eastern coastal parts of the Iberian Peninsula have long since been evidenced with the description and citation of several *P. officinale* subspecies (Boissier 1844, Willkomm and Lange 1880, Cadevall i Diars 1919–1923, Sennen 1913).

The first distinction from the Iberian populations in *P. officinale* was made with the description of *P. stenocarpum* Boiss. & Reut. ex Boiss., 1844 (Boissier 1844), which were grouped under this name, the populations being located in the centre of the Peninsula, characterised by the number of floral scapes (4 to 5), long leaf divisions of 3–4 inches in length (7.6–10.6 cm) and fructiferous raylets that triple the length of fruits, which are elliptic-ovate.

Subsequently, Willkomm and Lange (1880) cited P. officinale L. var. italicum (Mill.) DC. in Lam. & DC 1805 and *P. paniculatum* Loisel. (1807) in the Peninsula and these were added to encompass the dispersed populations in the central, northern and north-easterly parts of the Peninsula. Finally, these populations were assigned to *P. stenocarpum* (Cadevall i Diars 1919–1923).

Another name proposed was P. stenocarpum var. catalaunicum Pau in Sennen (1913) (nom. in sched.), but no description or herbarium specimens were provided. Reduron and Muckensturm (2008) gave a short Latin diagnosis but did not designate a lectotype.

Afterwards, *P. stenocarpum* was included as a subspecies of *P. officinale* (viz. P. officinale L. subsp. stenocarpum Font Quer 1950) and included all the populations in the eastern half of the Peninsula. Its morphological characteristics were summarised as: extended (up to 15 cm) and very narrow (from 0.3 to 2 mm) leaf segments, with between 10 and 64 umbel rays and a ratio of fructiferous pedicel length to fruit length from 0.5 to 1.5 (Font Quer 1954). Font Quer measured some of the populations that we have included in the present study and which have always been assigned the extreme value in the description of his study. Along with the subspecies officinale, they are the only two taxa in the group recognised in the Peninsula at this time. *Peucedanum paniculatum* and *P. longifolium* Waldst. & Kit. 1812 were reduced to subspecies of *Peucedanum officinale* due to their ternate leaves, which were divided into linear or linear-lanceolate segments by Frey (1989).

The last subspecies in the group, described in the Iberian Peninsula, Peucedanum officinale L. subsp. brachyradium García-Martín and Silvestre 1991 specifically in the province of Málaga (Spain), was indicated as an edapho-endemism on peridotites. That description derives from a population with only two individuals and is morphologically distinguished from the subspecies type and from *P. stenocarpum* by the characteristics of its inflorescence and by the dimensions of its fruit and pedicels. García Martín and Silvestre (1992) also suggested similarities with P. officinale subsp. longifolium.

The latest review undertaken of the genus for the Iberian Peninsula (Guillén and Laínz 2003) recognised only one species with two subspecies, viz. P. officinale subsp. officinale and P. officinale subsp. brachyradium. These authors included *P. stenocarpum* as a synonym of P. officinale subsp officinale, suggesting that there was variability and mixture within and between the populations.

## Materials and methods

While conducting fieldwork during the previous years, we located the population of an umbelliferous species in eastern spurs of the Serra Grossa mountain range, which lies in the southern part of the Valencian province (eastern part of the Iberian Peninsula). This species is always found at the bottom and top of rocky areas, on the edges of paths, always in cracks in rocks and on rocky soil. It is characterised by possessing ternatisect leaves, with linear and canaliculated leaf divisions; inflorescences with sessile lateral umbels and with few rays; white-petalled flowers; elliptic fruits with a strong dorsal compression and prominent dorsal ribs and winged marginal ribs, borne on short pedicels. As a result, we assigned it to the genus *Peucedanum* sensu stricto.

To further identify this taxon and compare all of the subspecies of *P. officinale* throughout its area of distribution, we reviewed the herbarium specimens of the genus deposited in the VAL and MA herbaria. We studied all the bibliography about the *P. officinale* subspecies cited and described in the Iberian Peninsula and the monograph on the genus by Frey (1989). For this monograph, 2,500 herbarium sheets from all over Europe were consulted and measurements were taken from 200 of them. For the subspecies P. officinale subsp. brachyradium, the measurements were provided in the description article (García Martín and Silvestre 1992).

**Figure 1.**
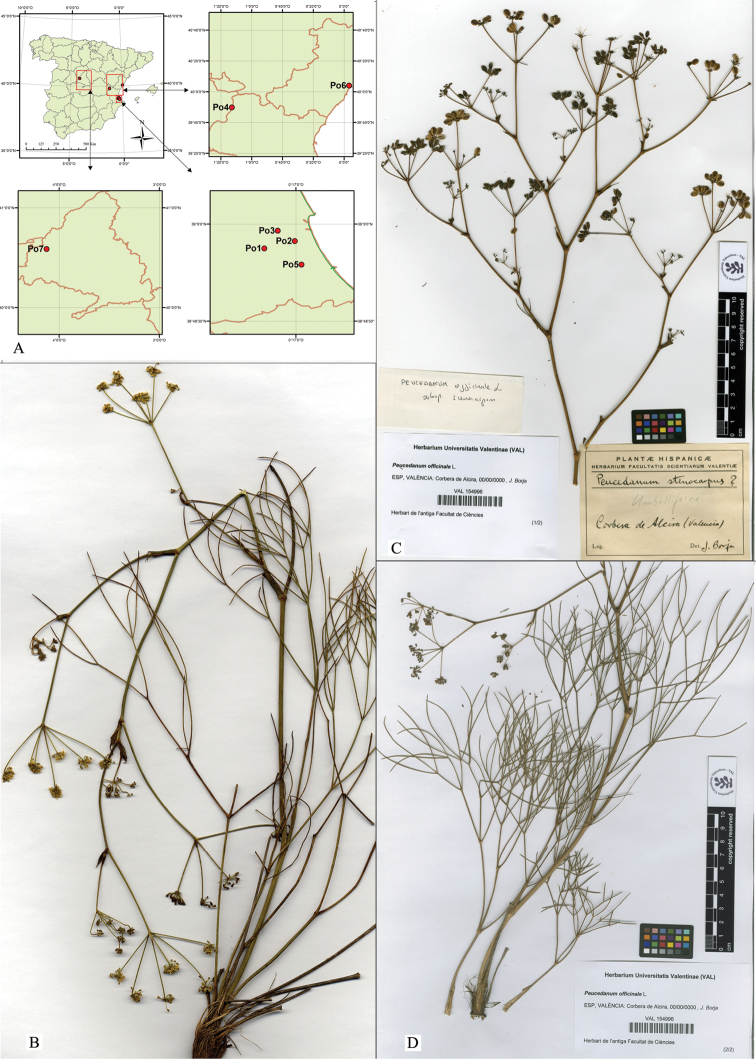
**A** Maps of studied populations **B**P. officinale subsp. album. Spain. Valencia: Alzira. Serra de la Murta, 30SYJ2934; on rocky outcrops, 25-IX -2011; J. Martínez-Fort (VAL 223162!) **C–D**P. officinale subsp. album. Spain. Valencia: Corbera de Alzira, 30SYJ23; J. Borja (VAL 154996!).

We noted that the population under investigation here had been identified to date as *P. stenocarpum*, widely considered as a synonym of P. officinale subsp. officinale, despite the fact that it has a series of morphological characters that are not accommodated within any of the subspecies of *P. officinale* (Figures 1B–D). For its characterisation and comparison, measurements of the plants were taken and the morphological ratios calculated for habit, leaves, inflorescences and fruits (Table 2, Suppl. material 1: Figure S1). The specimens belong to the located population in Carcaixent, as well as other closer populations located all along the coastline of the Valencian region (in Benicasim, Tavernes de la Valldigna, Alzira, Xeresa) (Figure 1A) with the same series of morphological characteristics. Hereafter we referred to this new studied subspecies as P. officinale subsp. album. To complete the results, we compared these populations with a population assigned to the subspecies type. We selected those populations located further inland, specifically between the provinces of Cuenca and Valencia, which correspond to P. officinale subsp. officinale. The series of populations, studied in the field, are provided in Table 1.

**Table 1.** Field populations (V Valencia, Cu Cuenca, Cs Castellón, M Madrid).

Population	Mountain range	Province/Towns	Local name	YWGS84 coordinates
**Po1**	Extreme north, Serra Grosa	V: Carcaixent, Alzira	Hort de Soriano-Font de la Parra, Molló de Miramar	39°3.88'N, 0°23.694'W
**Po2**	Spurs, Sierra de Corbera	V: Llaurí, Favara, Tavernes de la Valldigna	Font de la Granata, Pic Masalari	39°5.43'N, 0°17.238'W
**Po3**	Spurs, Sierra de Corbera	V: Alzira, Corbera, Llaurí	Serra de la Murta, Creu del Cardenal	39°7.61'N, 0°20.856'W
**Po4**	Sierra Picarcho	V: Tuejar, Cu:Talayuelas	Umbría del Picarcho	39°49.83'N, 1°13.002'W
**Po5**	Serra Mondúver	V: Gandia, Xeresa	Cima Mondúver	39°0.45'N, 0°15.834'W
**Po6**	Desert de les Palmes	Cs: Benicasim	La Comba	40°3.94'N, 0°3.144'E
**Po7**	South, Sierra de Guadarrama	M: El Escorial	El Escorial	40°35.09'N, 4°7.62'W

**Table 2.** Measured characters and calculated ratios.

Character				Units	Code
**Stem**	Size		Plant height	m	T1
	Ramification		First ramification height	m	T2
			T1/T2 ratio		T3
	Diameters		Diameter at the base	cm	T4
			Diameter at the first ramification	cm	T5
			T4/T5 ratio		T6
**Leaves**	Basal rosette	Blade	Leaf length, including petiole	mm	H1
			Number of leaf divisions (ternate)		H2
			Leaf width at the base of the last division	mm	H3
			Leaf width at the apex of the last division	mm	H4
			Last leaf division length	mm	H5
			H5/H3 ratio		H6
			H4/H5 ratio		H7
			Angle between contiguous leaf divisions of the last ternate	degrees	H8
			Angle between lateral leaf divisions of the last ternate	degrees	H9
		Petiole	Length	mm	H10
			Diameter at its basis	mm	H11
			Diameter before first division (ternate)	mm	H12
	First on stem from the basal rosette	Blade	Number of leaf divisions (ternate)		H13
			Last leaf division length	mm	H14
		Petiole	Length	mm	H15
			Diameter of the petiole at its basis	mm	H16
			Diameter before the first division (ternate)	mm	H17
	Last on stem	Blade	Number of leaf divisions (ternate)		H18
		Petiole	Length	mm	H19
			Diameter before the first division (ternate)	mm	H20
			Diameter at its basis	mm	H21
		Sheath	Sheath length	mm	H22
**Inflorescence**	Umbel		Bract length	mm	I1
			Bract width	mm	I2
			Number of bracts		I3
			I1/I2 ratio		I4
			Number of rays		I5
			Length of internal rays	mm	I6
			Length of external rays	mm	I7
	Umbellule		Number of raylets		I8
			Length of internal rays	mm	I9
			Length of external rays	mm	I10
			Number of bracteoles		I11
**Fruit**	Size		Length	mm	F1
			Width	mm	F2
			Wing width of the marginal ribs	mm	F3
	Shape		F3/F2 ratio		F4
			F1/F2 ratio		F5
	Raylet		Fructiferous raylets length	mm	F6
			F1/F6 ratio		F7
			F6/F1 ratio		F8
			F2/F6 ratio		F9

Measurements were taken in the field and in the laboratory and always carried out with fresh plants. We used a CD-20DCX digital vernier caliper and a metal tape measure and saved measurements on spreadsheets and databases. We created the figure of P. officinale subsp. album using the holotype VAL 223161 to scan, as well as from the photos taken in the field with a Canon EOS 550D camera of inflorescences and fruits. Other photos were taken with a Leica stereomicroscope MZ 9.5 and a Leica DFC 320 digital camera of the cross-sectional cuts of fruits and leaf details. We obtained the flowering and fructification data while conducting fieldwork and according to the date of the specimens and phenologies from the consulted herbarium sheets. All the other figures are photos of herbarium sheets and pictures of scanned herbarium sheets. We compared and statistically analysed the taken measurements with the Statgraphics Centurion XVI software. For morphological characters, we followed the standardised terminology of Kljuykov et al. (2004). For the taken measurements, we calculated the range obtained from their mean +/- standard deviation. The extreme values beyond this range are in brackets in the tables.

Taking measurements was limited by the small proportion of individuals which were flowering or under fructification in all the populations; indeed, flowering and fructification did not even exceed 4% of the individuals in the largest populations. In the specimens that presented no flowering, measurements were taken from the basal rosette leaves. In all, we took measurements from 31 specimens, of which 19 belonged to populations of the newly proposed taxon and 12 to the type subspecies. In all, 1,316 measurements and ratios were taken and calculated.

### Revised herbarium material

The examined herbarium specimens’ material at VAL and MA are listed below.

***Peucedanum longifolium* Waldst. & Kit.**

YUGOSLAVIA. **Dalmatia**: Montes Biokovo, in rupibus calcareis sub cacumine montis Sv. Jure supra opp. Makarska, 1600 m alt. 29-VII-1979, F.Cernoch (MA 357267); ídem (MA 310966)

***Peucedanum officinale* L.**

BULGARIA. **Regio Sophiensis**: distr. urb. Sophia, inter fruticeta supra, 20-IX-1979. N. Andreev, Z. Cerneva & P. Gerginov (MA 309655)

GERMANY. **Maingebaeit**: Würzburg, woodland between Gerbrunn and Kottendorf, 00-VII-1881, G. Evers (MA 713330)

SPAIN. **Aragón**:(MA 88547); Palau (MA 88581). **Alava**: Labastida, Salinillas de Buradón, 12-X-1990, P.M. Uribe-Echebarria & P. Urrutia (MA 523402); Labastida, Salinillas de Buradón, cerro calizo con matorral mediterráneo, 30TWN1320, 5-IX-1998, P.M. Uribe-Echebarría (VAL 144623); Labastida, Salinillas de Buradón, 02-X-1997, M. L. Gil Zúñiga (MA 616760); ídem (VAL 106191) **Albacete**: Molinicos, valle del río Mundo, entre Mesones y la fuente de la Plata, 30SWH587602, 900 m alt., 18-VIII-2002, M.J. Tohá & V.J. Arán (MA 703748); Molinicos, Valle del río Mundo, entre Mesones y la Fte. de la Plata, Laderas calcáreas con Cinar, 30SWH587602, 18-VIII-2002, V.J. Arán & M.J. Tohá (VAL 144146). **Barcelona**: Berga a Labaello, 16-VII-1911, Fre. Sennen (MA 88550); Al lado de la Ermita de S. Jerónimo, IX-1914, Caballero (MA 88584); Montserrat, IX-1905; Marcet (MA 88545). **Castellon**: Benicàssim (La Plana Alta), La Comba, 31TBE43, 07-IV-1990, J. Tirado & C. Villaescusa (VAL 26141); La Pobla Tornesa (La Plana Alta), Bartolo cresta, 31TBE44, 17-IX-1989, J. Tirado & C. Villaescusa (VAL 26140). **Cuenca**: Huete, hacia Garcinarro, valle del arroyo de Valquemado, 13-IX-2003, V. J. Arán & M. J. Tohá (MA 711491); Huete, hacia Garcinarro, valle de arroyo de Valquemado, 10-X-2004, V.J. Arán (MA 751028); Huete, hacia Garcinarro, valle del arroyo de Valquemado, al pie de cerros yesosos, 30TWK2449, 6-VIII-2005, V. J. Arán (VAL 179661); Huete, hacia Garcinarro, valle del arroyo de Valquemado, al pie de cerros yesosos, 30TWK245490, 13-IX-2003, V. J. Arán & M. J. Tohá (VAL 149187); Talayuelas, VII-1979, G. Mateo (VAL 110252); Talayuelas, X-1980, G. Mateo (VAL 110251). **Gerona**: Maçanes, 80 m alt., Font Quer, 12-X-1948 (MA 152333); ídem (MA 382969); Pyrénées à Gombreny, coteaux calcaires, 900 m alt., VIII-1913 (MA 88562), ídem (MA 88561), ídem (MA 88560). **Huesca**: Ayerbe, 600 m alt., 31-VIII-1973, A. Segura Zubizarreta (MA 359384); Arro, 26-IX-1979, P. & G. Montserrat (MA 357236); ídem (MA 311455). **Lérida**: La Granadella (Garrigues), hacia El Solerás, pr. riera de Vall de les Olives, junto a la carretera, 31TCF0486, 365 m alt., 08-IX-2008, V.J. Arán & M.J. Tohá (MA809441); ídem (VAL 196084). **Logroño**: Briones, Monte Lara, 1925, Hno. H. Elias (MA 88558). **Madrid**: Chozas, Cutanda, IX, (MA 88552); Entre Villalba y las Zorreras, en la Sierra del Guadarrama, 08-IX-1947, Rivas Goday & C. Pérez (MA 152463); ídem (MA 204879); ídem (MA 382966); Guadarrama, IX-1841, Reuter (MA 88577); Escorial, Graells (MA 720356); Guadarrama, J. Isern. (MA 720260). **Pontevedra**: Santa Maria de Oya, 10 m alt., 20-VIII-1983, S. Silvestre (MA 316210). **Salamanca**: Saucelle, 16-VIII-1978. F. Amich (MA 309660). **Tarragona**: Sant Carles de la Ràpita, Serra de Montsià, 450 m alt., 01-IX-1999, V. J. Arán & J. Masip (MA 631766); Sant Carles de la Ràpita, Serra de Montsià, Font de Burgà, hacia el SE, laderas soleadas sobre la fuente, entre el matorral calcícola. 31TBF9301, 1-VII-1999, Arán & Masip (VAL 41688). **Teruel**: Cantavieja, hacia Mirambel, 30TYK29, 4-IX-1993, Fabregat & López Udias (VAL 81745); Olba, IX-1894 (MA 88548); Castellote, alrededores de las Cuevas de Cañart, 11-IX-1991, C. Fabregat (MA 502852); ídem (VAL 75992); Olba, Caserío de la Berdeja, Ribazos. 30TXK9844, 25-IX-2004. S. López Udias & C. Fabregat (VAL 204090); San Agustín, valle del Mijares, hacia Rubielos de Mora, 30TXK9445, 4-IX-2004, G. Mateo (VAL 151501); Villarluengo, barranco de los Degollados, márgenes de la carretera, 30TYL00, 11-IX-1993, Mercadal (VAL 81675). **Valencia**: Ayora, La Hunde, 30SXJ52, 00-VIII-1981, J. B. Peris (VAL 17838); Bicorp, Cuesta de la Caruma, 25-VIII-1915, C. Vicioso (MA 88549); Corbera de Alcira, 30SYJ23, J. Borja. (VAL 154996); Serra de Corbera, 30SYJ23, 00-X-1944, J. Borja (VAL 117840), Favara: Serra de Corbera, 30SYJ33, 5-IX-1986, G. Mateo & al. (VAL 117826); Sinarcas, 30SXK50, 6-VII-1992, García Navarro (VAL 105040); Sinarcas, Peña del Rayo, 30SXK50, 12-IX-1989, García Navarro, (VAL 102949); Tuéjar (Serrans), Altos del Picarcho, rodenos, 30SXK5311, 12-IX-2004, C. Torres Gómez, G. Mateo & J. Fabado (VAL 217597); Tuéjar a Talayuelas, umbría del Picarcho, 30SXK51, VIII-1980, G. Mateo (VAL 110250); Tuéjar (Serrans) Altos del Picarcho, rodenos, 30SXK5310, 24-IX-2005, C. Torres Gómez (VAL 216649). **Zamora**: Muelas del Pan, 13-VIII-1978, E. Rico (MA 309659); Río Esña, Riberos del pantano de Ricobayo, 00-VII-1972, Rivas Goday & Ladero (VAL 117836). **Zaragoza**: El Frasno, 30TXL26208190, 2-IX-1995, A. Martínez (VAL 216135); Moncayo, 3-VIII-2000, Vicioso (VAL 180380); Torrero, 00-VI-1947, P. Capell S.J. (VAL 180379).

FRANCE. **Pyrénées-Orientales**. Conflent. En allant de Ille-sur-Têt à Montalba a 2 km env. de Montalba, 02-X-1970. J. Vivant (MA 357223); **La Garde Freinet**, an der D. 48 nörlich La Trémoulêde,, 4-X-1963 (MA 626021); **Languedoc-Roussillon**, Aude, sur le versant nord du col d´Extrème entre Villeneuve-des-Corbières et Tuchan, 15-IX-2004. Philippe Rabaute. (MA 802614); **Cher**: IX-1890, A. Le Grand (MA 88575)

HUNGARY. Bács Bodrog, Bezdan, 10-IX-1909, J. Prodan (MA 88576)

***Peucedanum paniculatum* Loisel.**

FRANCE. **Haute Corse**: Castagniccia, Col di Bigorno, 10-IX-1996, J. Lambinon (MA 628116); Ghisoni, 17-VIII-1899, R. Rotges (MA 88546); Ghisoni, Maquis peu touffu, 10-VIII-1929, Dr. C. Gabrel (MA 425140); Massif du Tenda, Col di Bigorno, mun. Bigorno, 07-VIII-1996, L. Serra & A. Bort (MA 623316).

### DNA extraction, amplification and sequencing

We made a genetic comparison of the ITS regions of ribosomal DNA between the populations of P. officinale subsp. officinale and P. officinale subsp. album, by extending the field sampling to one of the populations close to the classical location where *P. stenocarpum* had been described, El Escorial (Madrid) (Population Po7 in Figure 1 and Table 1). Using fragments of the basal leaves and shoots obtained from the seeds collected in the field, we extracted total DNA with the Plant DNA kit of Omega Bio-Tek, following the manufacturer’s instructions. To amplify the ITS regions of ribosomal DNA, we used oligonucleotides ITS5 and ITS4 (White et al. 1990) and the MBL Taq Polymerase kit of Molecular Biology Laboratory SL. Sequencing was done by MACROGEN using these same universal primers. The extraction process of the DNA extracted from the leaves collected in the field was complicated by the presence of metabolites, but was much easier to perform on the shoots of germinated seeds. We extracted DNA and sequenced the ITS region of ribosomal DNA from four populations (Table 3).

**Table 3.** The populations measured and the DNA samples taken. The details of these populations are shown in Table 1 and Figure 1A.

Populations		Number of plants	
Species	Population (Table 1)	Measurements taken	DNA sample
P. officinale subsp. album	Po1	4	
	Po2	4	
	Po3	9	
	Po5		1
	Po6		1
	Po8	2	
P. officinale subsp. officinale	Po4	12	1
	Po7		1

The obtained sequences were aligned with CLUSTALW from Bioedit 7.2.5. (Hall 1999). We aligned the consensus sequence obtained with BLASTn (Altschul et al. 1990) to obtain the genus *Peucedanum* sequences, with which a final set of sufficiently long sequences was used to be able to compare them with our sequence (Suppl. material 2: Table S1).

The pairwise genetic distances between sequences were calculated with MEGA, version X (Kumar et al. 2018) with 10,000 bootstraps replicates and gamma distribution (shape parameter = 0.7). The employed model was TN93+G, available in MEGA and amongst the best models obtained previously with JmodelTest and the Bayesian Information Criterion (BIC) (Darriba et al. 2012).

## Results and discussion

**Figure 2.**
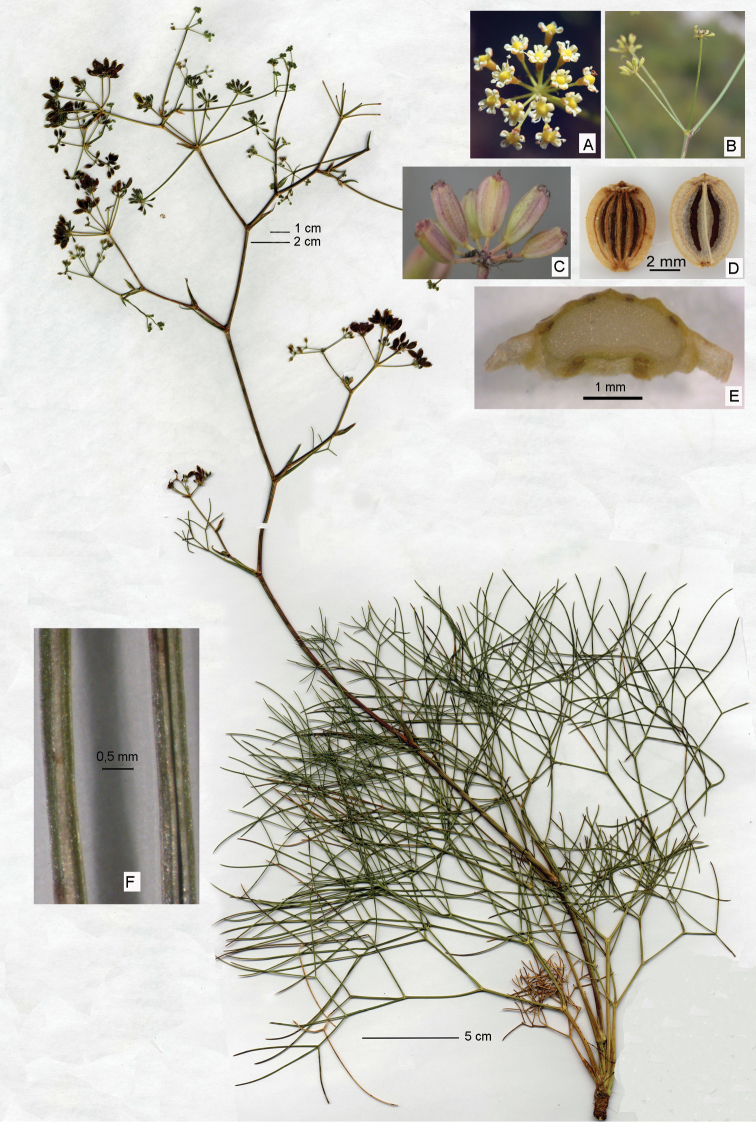
Holotype of Peucedanum officinale subsp. album from Tavernes de la Valldigna, Valencia (VAL 223161!). Photos taken in the field: **A** flowers **B** sessile umbel with three rays **C** fruits. Photos taken with a stereomicroscope **D** Mericarp **E** fruit cross-section **F** leaf underside and bundle.

By taking the morphological differences observed in leaves, inflorescences and fruits, its habitat and distribution in the humid and sub-humid thermomediterranean bioclimatic types as a basis, we distinguished P. officinale subsp. album as a new subspecies, after its comparison with all the revised herbarium specimens and data provided in the bibliography (Table 4). Compared to the nominated subspecies, with which it has contact in its distribution, it is easily distinguished by the white colour of the petals (Figure 2A), its inflorescences without dominant umbels and umbels with scarce rays and sometimes sessile and with canaliculated leaflets. There is a difference in the subspecies type that possesses inflorescences with dominant umbels of a greater number of rays and with flat leaflets (Figs 4 A–B). P. officinale subsp. album can be further distinguished from subspecies paniculatum, which is restricted to the islands of Corsica and Sardinia, by its inflorescences, which are much more branched and paniculated, with many rayed umbels and fruit pedicels that are three times the length of the fruit. The subspecies brachyradium has very limited distribution being an edapho-endemic species on peridotite of the province of Malaga. It is distinguished by its greater bearing, inflorescence with more rays and fruit pedicels equal in length to the fruit.

**Table 4.** Comparison between the results obtained from the field measurements that we took with data reported in other studies about *P. officinale* subspecies: subsp. officinale (Figure 4C), subsp. longifolium (Figure 4E), subsp. paniculatum (Figure 4D). Data provided in the monograph of the genus (Frey 1989) and in the description of P. officinale subsp. brachyradium (García Martín and Silvestre 1992).

Origin of data	Field data	Frey 1989				García Martín and Silvestre 1992
Organ	* album *	* officinale *	* stenocarpum *	* longifolium *	* paniculatum *	* brachyradium *
Habit (cm)	(24)41–98 (130)	60–140(200)	120	(37)70–150(360)	60–100 (120)	250
Leaf	canaliculated	flat	flat	crested	canaliculated	canaliculated
Last division basal leaf length (mm)	(31.3)47.9–83.7(94.4)	(20)30–60(100)	(27)40–85(95)	(13)35–80(165)	(17)24–35(53)	20–55
Last division basal leaf width (mm)	0.3–0.6(0.7)	(0.7)1–2(2.7)	(0.9)1–1.8(2)	(0.5)1–1.8(2.3)	0.5–0.8(1)	0.8–1.3(2.5)
Bracts of umbels	0–1(2)	(0)1–10	0–1(4)	(0)1–10	0–1(2)	(0)1–2(5)
Umbel rays	(3)5–9(12)	(12)17–35(58)	(18)20–37(41)	(14)16–32(49)	9–14(17)	12–18
Umbel ray length (mm)	(2.8)8–43.8(83.7)	(11)30–85(150)	(40)49–90(100)	(18)25–75(108)	(24)30–60(90)	15–40
Umbellule raylets	(7)10–16(18)	(14)18–35(50)	(12)31–30(34)	(7)15–36 (44)	??	9–16
Colour of petals	white	yellow	yellow	yellow	yellowish	yellow
Fruit length (mm)	(5.1)5.8–6.9(7.1)	5.5–9	05–7	5.5–7	5.5–6	7.6–9.7
Raylet length/Fruit length	(0.3)0.5–0.8(1)	2–6	1.5–4	1	1–3	2

Regarding the genetic results obtained, the ITS sequences of the studied specimens that belong to the populations of Xeresa, Benicasim, Tuejar and El Escorial were identical to one another. The length of the obtained consensus sequence was 603 base pairs. It is deposited in GenBank http://www.ncbi.nlm.nih.gov/GenBank). The accession number is KP681852.

This sequence is identical to all the *P. officinale* sequences in GenBank and has a comparable length when using sets of sequences with both 319 bp and 640 bp (with gaps) (Suppl. material 2: Table S1). In comparison, the distance between *P. officinale* and the other species of the *Peucedanum* s.str. varied from 0.003 with *P. gallicum* Latour. and 0.211 with *P. sandwicense* Hillebr. The distance with *P. gallicum* is very small, however it is a species clearly accepted. As the distances are so small between near species, the analysis has not allowed separation genetically at the subspecific level. This supports the intraspecific range in P. officinale for the subspecies album.

The briefness and the small number of characteristics that have been analysed in the studies have meant that our determination of these populations was complicated. With the exhaustive comparison that we have carried out, we have been able to identify the characteristics (Table 4) that distinguish these coastal populations as another subspecies. In many of the descriptions and determination keys, leaf morphology was stressed: the length, width and angle of the last-order leaf divisions; and some fruit characters or the number of umbel rays were also used to classify the different subspecies. According to the measurements and observations made in the field, we do not consider the length and width of the last leaf division to display good characters for distinction, as it could have already varied in the same population according to the environmental conditions where the plant grows.

Conversely, the leaflets shape characteristics of the last divisions; flat, folded-crested or canaliculated and the shape of inflorescences: with terminal, pedunculate umbels with 17–35 rays that are 30–85 mm long or sometimes with panicled inflorescences or with umbels, some of which are sessile and lateral, with 5–9 rays measuring 8–43 mm, were determining factors for separating the subspecies. Thus, the *album*, *brachyradium* and *paniculatum* subspecies share the combination of canaliculated leaflets and panicled inflorescences or with sessile umbels and fewer rays; as opposed to the *officinale* and *longifolium* subspecies, which have flat or grooved leaflets and inflorescences with terminal umbels and more rays.

These values obtained in the field have been used for the description of P. officinale subsp. album (Table 5). Of them all, we obtained statistically significant differences with a 95% confidence level between the two subspecies album and *officinale* in the following characters (Table 6). These parameters are the number of rays of the umbels and umbellules (raylets), the length of the fruit and its peduncle and the proportion in the leaves between length and width and angles of separation between the last leaf divisions (leaflets).

**Table 5.** Values obtained from the measurements taken in the field.

Code (Table 2)	Character (Table 2)	P. officinale subsp. album	P. officinale subsp. officinale
**Stem**			
**T1**	Plant height	(0.24)0.41–0.98(1.3) m	(0.27)0.42–1.26(1.74) m
**T2**	First ramification height	(0.07)0.14–0.39(0.5) m	(0.12)0.15–0.52(0.73) m
**T3**	T1/T2 ratio	(1.6)2–3.5(3.9)	1.3–3.6(4.6)
**T4**	Diameter at the base of stem	2.1–4.6(6.3) mm	(2)2.7–6.1(7.3) mm
**T5**	Diameter at the first ramification	2–4.5(6.2) mm	(1.7)2.3–6.4(8.6) mm
**T6**	T4/T5 ratio	(0.8)0.9–1.1(1.2)	0.9–1.2(1.3)
**Basal rosette leaf**			
**H1**	Leaf length, including petiole	(190)257–538(630) mm	(280)335–505(570) mm
**H2**	Number of leaf divisions (ternate)	4–5(6)	4–5(6)
**H3**	Leaf width at the base of the last division	(0.4)0.5–0.8(0.9) mm	(0.5)0.6–1(1.1) mm
**H4**	Leaf width at the apex of the last division	0.3–0.6(0.7) mm	(0.6)0.7–1.1(1.4) mm
**H5**	Leaf division length	(31.3)47.9–83.7(94.4) mm	(45.6)47.6–76.7(85.6) mm
**H6**	H5/H3 ratio	(50.5)70.3–125.8(166.2)	(57.1)62–96.3(108)
**H7**	H4/H5 ratio	(3.2)4.1–10.7(17.2) ×10^-3^	(10.2)11.4–18(19.4) ×10^-3^
**H8**	Angle between contiguous leaf divisions of the last ternate	(30)32–66(90) degrees	(30)31–45 degrees
**H9**	Angle between lateral leaf divisions of the last ternate	(45)111–180 degrees	45–138(180) degrees
**H10**	Length petiole leaf	(42)53–171(250) mm	(55)87–197(240) mm
**H11**	Diameter at its basis petiole leaf	1.5–2.8(3.4) mm	(2.1)2.2–3.4(3.7) mm
**H12**	Diameter before first division (ternate) leaf	(1.5)1.9–3.5(4.4) mm	(2.7)2.8–4.4(5.2) mm
Cauline leaves: **First on stem from the basal rosette leaf**			
**H13**	Number of leaf divisions (ternate)	3–4(5)	(2)3–5
**H14**	Last leaf division length	(1.8)6.5–36.4(40.8) mm	5–88.8(147.3) mm
**H15**	Length	(1.2)4.3–61.1(87.3) mm	9.5–73.4(95.2) mm
**H16**	Diameter of the petiole at its basis	(0.8)0.9–2.2(2.6) mm	(0.7)1.2–2.9(3.1) mm
**H17**	Diameter before the first division (ternate)	(0.8)1–2.2(2.7) mm	(0.7)0.9–4.2(5.8) mm
Cauline leaves: **Second on stem leaf**			
**H18**	Number of leaf divisions (ternate)	1–3(4)	2–4
**H19**	Length	3.2–73.8(120) mm	41–67.5(70) mm
**H20**	Diameter before the first division (ternate)	(0.5)0.7–1.7(2.1) mm	1–2.4(2.5) mm
**H21**	Diameter at its basis	0.9–2 mm	1–2.1(2.2) mm
**H22**	Sheath length	(2)6.5–22.6(28.3) mm	22–29.5(31.4) mm
**Umbel**			
**I1**	Bract length	(0.6)0.7 mm	(0.5)0.6 mm
**I2**	Bract width	1.1(1.3) mm	1.7(1.8) mm
**I3**	Number of bracts	0–1(2)	0–2(3)
**I4**	I1/I2 ratio	3.5–7.6(9.1)	(3.2)4.3–10.5(12)
**I5**	Number of rays	(3)5–9(12)	(13)15–32(35)
**I6**	Length of internal rays	(2.8)4–24.3(42.6) mm	(5)6.2–24.3(29.7) mm
**I7**	Length of external rays	(15.7)17.7–52.6(83.7) mm	(21.4)23.5–71.2(82.7) mm
**Umbellule**			
**I8**	Number of raylets	(7)10–16(18)	(7)12–24(29)
**I9**	Length of internal rays	0.2–6.5(13.1) mm	2.1–21.7(35.3) mm
**I10**	Length of external rays	0.3–17.6(35.1) mm	5.2–43.6(71.2) mm
**I11**	Number of bracteoles	(4)6–9	(6)7–9(10)
**Fruit**			
**F1**	Length	(5.1)5.8–6.9(7.1) mm	(3.7)5.9–8.1(8.5) mm
**F2**	Width	(2.6)3.3–4.7(5) mm	(2.6)3.3–4.4(4.8) mm
**F3**	Wing width of the marginal ribs	(0.2)0.4–1(1.1) mm	(0.4)0.6–1.1 mm
**F4**	F3/F2 ratio	(0)0.1–0.3	0.1–0.2
**F5**	F1/F2 ratio	(1.2)1.3–1.9(2)	(1.4)1.7–2(2.1)
**F6**	Fructiferous raylet length	(1.8)2.8–5.3(6.1) mm	(3.8)5.8–10.4(11.7) mm
**F7**	F1/F6 ratio	(1)1.2–2.3(3.2)	(0.6)0.7–1(1.2)
**F8**	F6/F1 ratio	(0.3)0.5–0.8(1)	(0.8)1–1.3
**F9**	F2/F6 ratio	0.6–1.6(2.8)	(0.3)0.4–0.5(0.7)

**Table 6.** Values with statistically significant differences. Code refers to Table 2.

Code	Organ	subsp. album	subsp. officinale
**I5**	Number of rays (u.)*	(3) 5–9 (12)	(13) 15–32 (35)
**I8**	Number of raylets (u.)*	(7) 10–16 (18)	(7) 12–24 (29)
**F1**	Fruit length (mm)*	(5.1) 5.8–6.9 (7.1)	(3.7) 5.9–8.1 (8.5)
**F6**	Fruit raylet length (mm) *	(1.8) 2.8–5.3 (6.1)	(3.8) 5.8–10.4 (11.7)
**F7**	Fruit length/ Raylet length *	(1) 1.2–2.3 (3.2)	(0.6) 0.7–1 (1.2)
**F8**	Raylet length/Fruit length **	(0.3) 0.5–0.8 (1)	(0.8) 1–1.3
**F9**	Fruit width/ Raylet length *	0.6–1.6 (2.8)	(0.3) 0.4–0.5 (0.7)
**H7**	Last basal leaf division: width/length***	(3.2) 4.1–10.7 (17.2) 10^-3^	(10.2)11.4–8 (19.4) 10^-3^
**H9**	Largest angle for external leaf divisions, last ternate (degrees) ***	(45) 111–180	45–138 (180)

* With more than 30 types of data. Differences obtained between the means with an ANOVA analysis; as normality or homoscedasticity was not met, the result was confirmed by the Kruskal Wallis test. ** With more than 30 data. Differences obtained between the means with an ANOVA analysis. *** With fewer than 30 data. If normality and homoscedasticity were met, the Mann-Whitney W (Wilcoxon) test was used.

## Taxonomic treatment

### Peucedanum officinale L. subsp. album

Taxon classificationPlantaeApialesApiaceae

Martínez-Fort & Donat-Torres subsp. nov.

007C468883D853498940A467673F00D6

urn:lsid:ipni.org:names:60479357-2

#### Diagnosis.

Peucedanum officinale L. subsp. album can be morphologically distinguished by the canaliculated leaflets, inflorescences without dominant terminal umbels, which are often sessile and lateral, with few rays (3) 5–9 (12), umbellules with (7) 10–16 (18) raylets, white-petalled flowers and fruits with a fructiferous raylet as long as or shorter than the length of the fruits.

#### Type.

Valencia, Tavernes de la Valldigna, eastern spurs of the Serra de les Agulles mountain range, on both ascents to Pic Massalari. 39,09°N, -0,284°W. 300 m alt. Rocky calcareous soil. 23-IX-2012. J. Martínez-Fort (**Holotype**: VAL 223161! Figure 2).

#### Description.

Perennial plant with stem (0.24) 0.41–0.98 (1.3) m high and 2.1–4.6 (6.3) mm in diameter at the base, branching from the lower 1/3–1/2, striate, glabrous. Basal leaves 4- or 5- (6-) ternate, triangular in outline, (190) 257–538 (630) mm in length; petioles cylindrical, (42)53–171(250) mm long, sheathing at the base; linear terminal leaflets, (31.3) 47.9–83.7 (94.4) mm × 0.3 to 0.6 (0.7) mm, canaliculate, length/width ratio of (50) 70–126 (166) range, angle between the closest divisions of the terminals (30) 32–66 (90) degrees and (45) 111–180 degrees between the outermost leaf divisions. Cauline leaves, decreasing in size towards the apex of stems, but with enlarged sheaths. The first leaf on stem from the basal rosette leaf 3–4 (5) times ternate, with terminal divisions of (1.8) 6.5–36.4 (40.8) mm of length and canaliculated. The uppermost ones reduced to the sheath.

Inflorescence without a primary or dominant umbel, umbels arranged along the axis of inflorescence, sometimes sessile and lateral. Umbels compound, rays (3) 5 to 9 (12), inner rays shorter (2.8) 4- 24.3 (42.6) mm, outer rays (15.7) 17.7–52.6 (83.7) mm, bracts 0 or 1 (2), linear-triangular, (0.6) 0.7× 1.1 (1.3) mm. Umbellules with (7) 10 to 16 (18) raylets, bracteoles (4) 6 to 9, linear, gradually widened towards base. Flowers hermaphroditic, sepals 5 triangular, inconspicuous; petals 5, white, inflexed tips; stamens 5, alternate; stylopodium conical, similar in length to the styles; styles parallel at anthesis, becoming divergent in fruiting. Fruits dorsally compressed, elliptical, size (5.1)5.8–6.9 (7.1) × (2.6) 3.3–4.7 (5) mm, apex slightly off emarginated; mericarps homomorphic; median and lateral prominent ribs, apex slightly off emarginated; marginal ribs prominently winged, wings (0.2) 0.4–1 (1.1) mm, wide; commissural vittae 2; vallecular vittae 4; commissure very broad, from wing tip to wing tip. Raylets (1.8) 2.8–5.3 (6.1) mm long. The raylets length/fruit length ratio ranging from (0.3) 0.5–0.8 (1).

#### Distribution and habitat.

**Figure 3.**
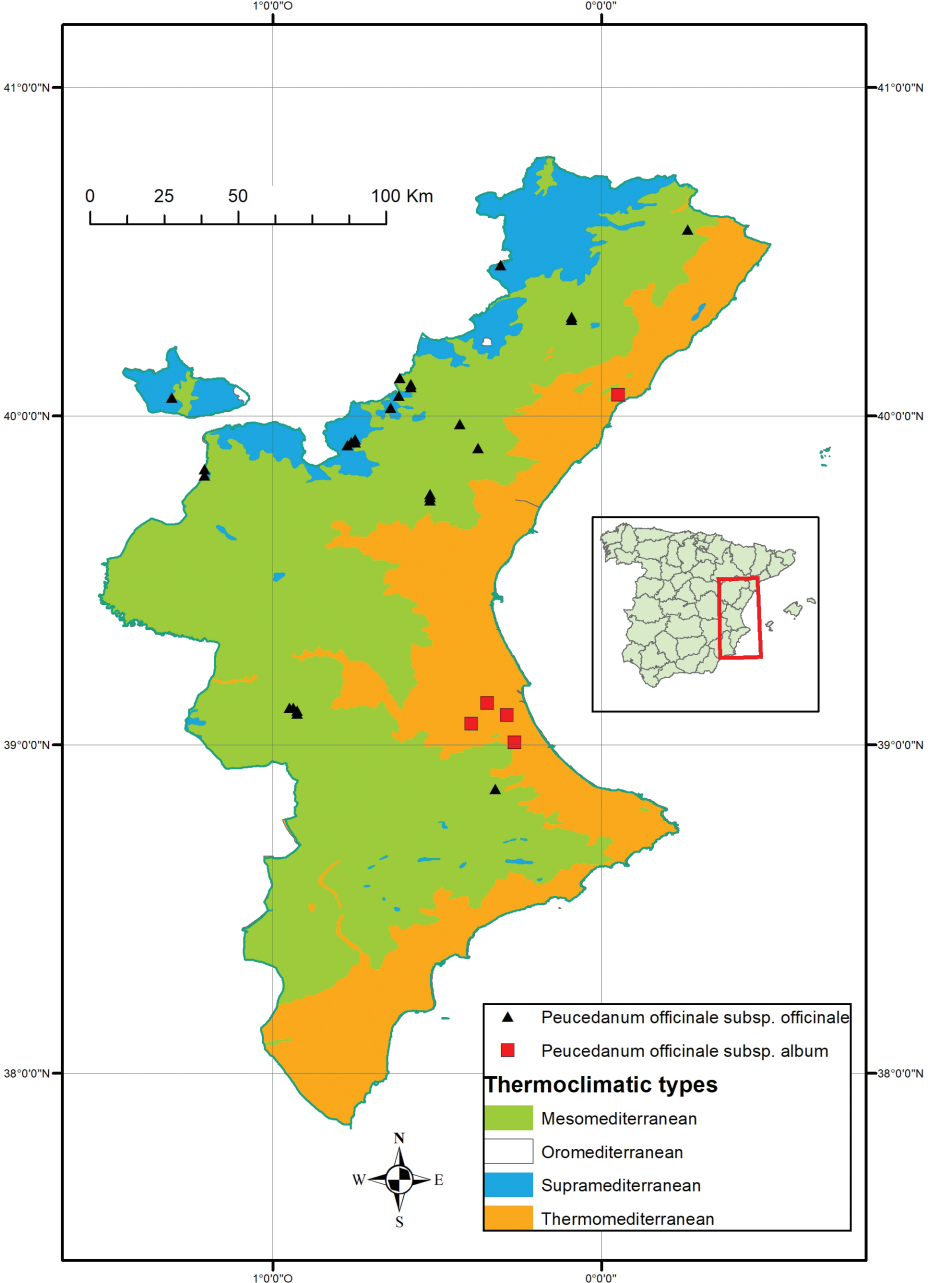
Map of Peucedanum officinale subsp. album (squares) and P. officinale subsp. officinale (triangles) on the Valencian Community region (Spain). The thermoclimatic bioclima types of the region are indicated in colour.

It is dispersed in the thermomediterranean sub-humid bioclima (Rivas-Martínez 2004) on rocky soils of limestone and sandstone at the base and crest of cliffs and rocky slopes. Contacting with wet fringes of *Rubio longifoliae-Quercetum rotundifolie* Costa, Peris and Figuerola (Costa et al. 1983), together with ash and arbutus. With abundant populations on the coastal and southern foothills of the Iberian system (Serra de les Agulles) and on the northern coastal foothills of the external Prebaetic system (Serra Grossa) (Figure 3).

#### Phenology.

Flowering July to October.

#### Paratypes.

SPAIN. Valencia: Alzira. Serra de la Murta, 30SYJ2934; on rocky outcrops, 25-IX-2011; J. Martínez-Fort (VAL 223162!, Figure 1B; Valencia: Corbera de Alzira, 30SYJ23; J. Borja (VAL 154996!; Figure 1C–D); Valencia: Favara, Serra de Corbera, 30SYJ33, 5-IX-1986; G. Mateo & al. (VAL 117826!).

##### Key to the subspecies of *Peucedanum officinale*

**Table:** 

1	Canaliculated leaflets (Figure 4B)	**2**
–	Non-canaliculated leaflets (Figure 4A)	**4**
2	Habit of up to 1 (1.3) m; petals white or pale yellow; fruit length < 7 mm	**3**
–	Habit of up to 1.5 m; petals yellow; fruit length > 7 mm	**subsp. brachyradium**
3	Umbels with 5 to 9 (12) rays, fructiferous raylets shorter than or equal to fruit length; petals white	**subsp. album**
–	Umbels with 9 to 14 (17) rays, fructiferous raylets longer than fruit length, up to 3 times the length; petals pale to medium yellow	**subsp. paniculatum**
4	Fructiferous raylets equal or shorter than fruit length; folded or crested (concave) leaflets	**subsp. longifolium**
–	Fructiferous raylets longer than fruit length; flat leaflets	**subsp. officinale**

**Figure 4.**
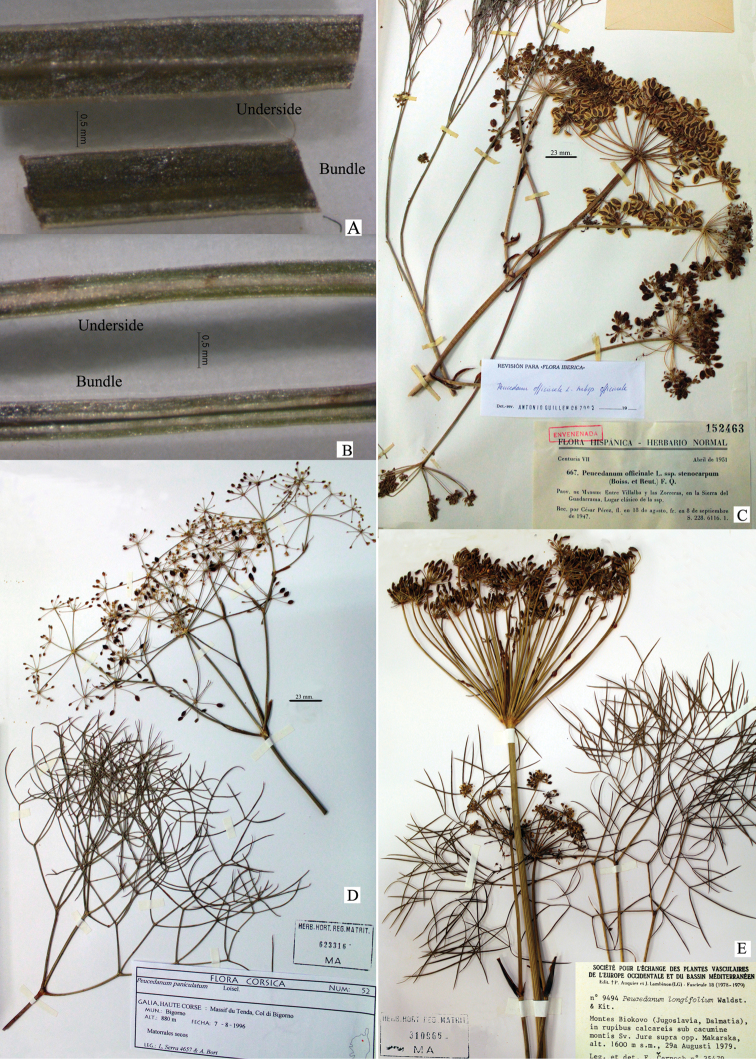
Types of bundle leaf **A** Flat-bundle leaflet of Peucedanum officinale subsp. officinale**B** Canaliculated-bundle leaflet of *Peucedanum officinale subsp. album*. A comparison of the other subspecies of *P. officinale***C**Peucedanum officinale subsp. officinale**D**P. officinale subsp paniculatum. **E**P. officinale subsp. longifolium. **Vouchers**: **C** C. Pérez. (MA 152463) **D** L. Serra & A. Bort, (MA 623316) **E, F** Cernoch, (MA 310966).

## Supplementary Material

XML Treatment for Peucedanum officinale L. subsp. album

## Supplementary materials

Supplementary material 1 Figure S1. Measured characters Data type: species data Explanation note: The figure shows the characters measured on the plants. https://binary.pensoft.net/file/328910This dataset is made available under the Open Database License (http://opendatacommons.org/licenses/odbl/1.0/). The Open Database License (ODbL) is a license agreement intended to allow users to freely share, modify, and use this Dataset while maintaining this same freedom for others, provided that the original source and author(s) are credited.Javier Martínez-Fort, Maela León, Maria P. Donat-Torres

Supplementary material 2 Table S1. Sequences obtained from GenBank to calculate pairwise genetic distances. Data type: molecular data Explanation note: Accession numbers of 85 ITS sequences downloaded from GenBank. The sequences from this study are in bold letters. https://binary.pensoft.net/file/328911This dataset is made available under the Open Database License (http://opendatacommons.org/licenses/odbl/1.0/). The Open Database License (ODbL) is a license agreement intended to allow users to freely share, modify, and use this Dataset while maintaining this same freedom for others, provided that the original source and author(s) are credited.Javier Martínez-Fort, Maela León, Maria P. Donat-Torres
