# Supplementary figures and images for: A new subspecies of Peucedanum officinale L. subsp. album (Apiaceae) from the eastern part of the Iberian Peninsula
Source: PhytoKeys. 2019 Sep 2;131:37–55. doi: 10.3897/phytokeys.131.32173 (PMC6733802; doi:10.3897/phytokeys.131.32173)

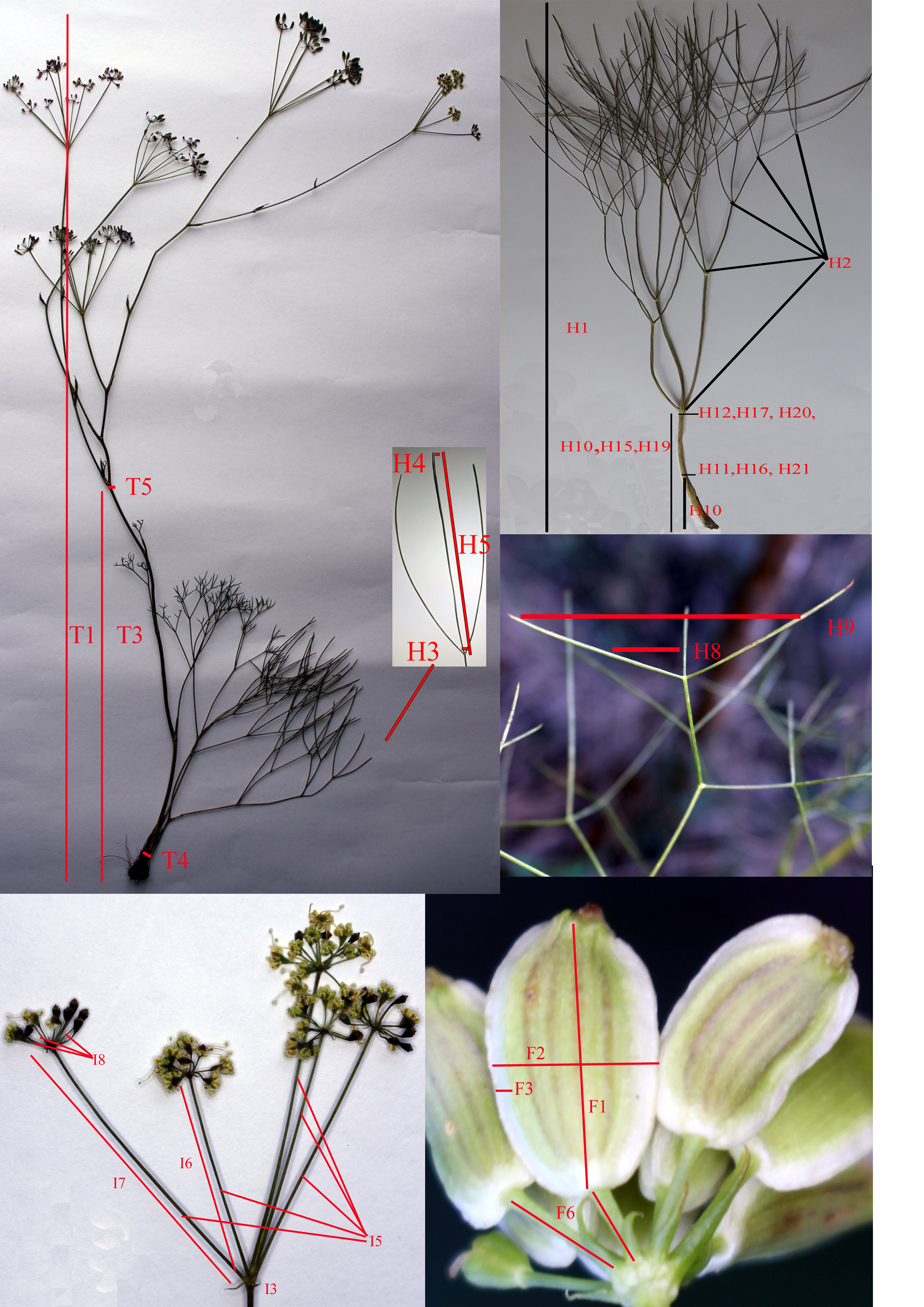

Supplement: Supplementary material 1 [file phytokeys-131-037-s001.jpg]
