# Supplementary material for: A new subspecies of Peucedanum officinale L. subsp. album (Apiaceae) from the eastern part of the Iberian Peninsula
Source: PhytoKeys. 2019 Sep 2;131:37–55. doi: 10.3897/phytokeys.131.32173 (PMC6733802; doi:10.3897/phytokeys.131.32173)
Supplement: Supplementary material 2 [file phytokeys-131-037-s002.docx]

**Table S1**. Sequences obtained from GenBank to calculate pairwise genetic distances. In bold letter sequence from this study.

| **Accesion** | **Organism** | **Accesion** | **Organism** |
| --- | --- | --- | --- |
| [AM408881](https://www.ncbi.nlm.nih.gov/nuccore/AM408881) | *Peucedanum angolense* | [KP278145](https://www.ncbi.nlm.nih.gov/nuccore/KP278145) | *Peucedanum multivittatum* |
| [KP682403](https://www.ncbi.nlm.nih.gov/nuccore/KP682403) | *Peucedanum austriacum* | [HQ269390](https://www.ncbi.nlm.nih.gov/nuccore/HQ269390) | *Peucedanum nebrodense* |
| [JF977803](https://www.ncbi.nlm.nih.gov/nuccore/JF977803) | *Peucedanum caespitosum* | [**KP681852**](https://www.ncbi.nlm.nih.gov/nuccore/KP681852) | ***Peucedanum officinale* subsp. *album*** |
| [JF977802](https://www.ncbi.nlm.nih.gov/nuccore/JF977802) | *Peucedanum caespitosum* | [KF160673](https://www.ncbi.nlm.nih.gov/nuccore/KF160673) | *Peucedanum officinale* |
| [KP682412](https://www.ncbi.nlm.nih.gov/nuccore/KP682412) | *Peucedanum cervaria* | [KX167724](https://www.ncbi.nlm.nih.gov/nuccore/KX167724) | *Peucedanum officinale* |
| [FJ385054](https://www.ncbi.nlm.nih.gov/nuccore/FJ385054) | *Peucedanum delavayi* | [KX167719](https://www.ncbi.nlm.nih.gov/nuccore/KX167719) | *Peucedanum officinale* |
| [EU418386](https://www.ncbi.nlm.nih.gov/nuccore/EU418386) | *Peucedanum delavayi* | [KX167720](https://www.ncbi.nlm.nih.gov/nuccore/KX167720) | *Peucedanum officinale* |
| [EU418388](https://www.ncbi.nlm.nih.gov/nuccore/EU418388) | *Peucedanum dissolutum* | [KP682404](https://www.ncbi.nlm.nih.gov/nuccore/KP682404) | *Peucedanum officinale* |
| [AM408885](https://www.ncbi.nlm.nih.gov/nuccore/AM408885) | *Peucedanum elegans* | [AF077896](https://www.ncbi.nlm.nih.gov/nuccore/AF077896) | *Peucedanum ostruthium* |
| [KP334195](https://www.ncbi.nlm.nih.gov/nuccore/KP334195) | *Peucedanum elegans* | [KF160674](https://www.ncbi.nlm.nih.gov/nuccore/KF160674) | *Peucedanum ostruthium* |
| [KP334196](https://www.ncbi.nlm.nih.gov/nuccore/KP334196) | *Peucedanum elegans* | [KP682405](https://www.ncbi.nlm.nih.gov/nuccore/KP682405) | *Peucedanum ostruthium* |
| [KP334197](https://www.ncbi.nlm.nih.gov/nuccore/KP334197) | *Peucedanum elegans* | [KP682407](https://www.ncbi.nlm.nih.gov/nuccore/KP682407) | *Peucedanum ostruthium* |
| [KP334198](https://www.ncbi.nlm.nih.gov/nuccore/KP334198) | *Peucedanum elegans* | [KP682410](https://www.ncbi.nlm.nih.gov/nuccore/KP682410) | *Peucedanum ostruthium* |
| [KP334199](https://www.ncbi.nlm.nih.gov/nuccore/KP334199) | *Peucedanum elegans* | [KF843810](https://www.ncbi.nlm.nih.gov/nuccore/KF843810) | *Peucedanum palimbioides* |
| [KP334200](https://www.ncbi.nlm.nih.gov/nuccore/KP334200) | *Peucedanum elegans* | [KF843811](https://www.ncbi.nlm.nih.gov/nuccore/KF843811) | *Peucedanum palimbioides* |
| [KP334201](https://www.ncbi.nlm.nih.gov/nuccore/KP334201) | *Peucedanum elegans* | [KX167987](https://www.ncbi.nlm.nih.gov/nuccore/KX167987) | *Peucedanum palustre* |
| [EU418385](https://www.ncbi.nlm.nih.gov/nuccore/EU418385) | *Peucedanum elegans* | [KX167986](https://www.ncbi.nlm.nih.gov/nuccore/KX167986) | *Peucedanum palustre* |
| [KF806571](https://www.ncbi.nlm.nih.gov/nuccore/KF806571) | *Peucedanum formosanum* | [GU190154](https://www.ncbi.nlm.nih.gov/nuccore/GU190154) | *Peucedanum parkinsonii* |
| [AM408882](https://www.ncbi.nlm.nih.gov/nuccore/AM408882) | *Peucedanum gallicum* | [DQ132871](https://www.ncbi.nlm.nih.gov/nuccore/DQ132871) | *Peucedanum praeruptorum* |
| [JF714990](https://www.ncbi.nlm.nih.gov/nuccore/JF714990) | *Peucedanum guangxiense* | [EU592009](https://www.ncbi.nlm.nih.gov/nuccore/EU592009) | *Peucedanum praeruptorum* |
| [JN603233](https://www.ncbi.nlm.nih.gov/nuccore/JN603233) | *Peucedanum hakuunense* | [KF806577](https://www.ncbi.nlm.nih.gov/nuccore/KF806577) | *Peucedanum praeruptorum* |
| [JF977795](https://www.ncbi.nlm.nih.gov/nuccore/JF977795) | *Peucedanum harry-smithii* | [KF806579](https://www.ncbi.nlm.nih.gov/nuccore/KF806579) | *Peucedanum praeruptorum* |
| [JF977786](https://www.ncbi.nlm.nih.gov/nuccore/JF977786) | *Peucedanum harry-smithii* | [EU418387](https://www.ncbi.nlm.nih.gov/nuccore/EU418387) | *Peucedanum rubricaule* |
| [JF977784](https://www.ncbi.nlm.nih.gov/nuccore/JF977784) | *Peucedanum harry-smithii* | [JX962353](https://www.ncbi.nlm.nih.gov/nuccore/JX962353) | *Peucedanum sandwicense* |
| [KP278144](https://www.ncbi.nlm.nih.gov/nuccore/KP278144) | *Peucedanum insolens* | [GU190153](https://www.ncbi.nlm.nih.gov/nuccore/GU190153) | *Peucedanum siamicum* |
| [AJ131343](https://www.ncbi.nlm.nih.gov/nuccore/AJ131343) | *Peucedanum japonicum* | [EU418389](https://www.ncbi.nlm.nih.gov/nuccore/EU418389) | *Peucedanum songpanense* |
| [BBAB697612](https://www.ncbi.nlm.nih.gov/nuccore/BBAB697612) | *Peucedanum japonicum* | [JF714989](https://www.ncbi.nlm.nih.gov/nuccore/JF714989) | *Peucedanum sp.* |
| [DQ270201](https://www.ncbi.nlm.nih.gov/nuccore/DQ270201) | *Peucedanum japonicum* | [AM408866](https://www.ncbi.nlm.nih.gov/nuccore/AM408866) | *Peucedanum sulcatum* |
| [RKX757776](https://www.ncbi.nlm.nih.gov/nuccore/RKX757776) | *Peucedanum japonicum* | [AM408884](https://www.ncbi.nlm.nih.gov/nuccore/AM408884) | *Peucedanum tauricum* |
| [RKX757777](https://www.ncbi.nlm.nih.gov/nuccore/RKX757777) | *Peucedanum japonicum* | [AY548216](https://www.ncbi.nlm.nih.gov/nuccore/AY548216) | *Peucedanum terebinthaceum* |
| [EU224273](https://www.ncbi.nlm.nih.gov/nuccore/EU224273) | *Peucedanum japonicum* | [AM408886](https://www.ncbi.nlm.nih.gov/nuccore/AM408886) | *Peucedanum terebinthaceum* |
| [EU224274](https://www.ncbi.nlm.nih.gov/nuccore/EU224274) | *Peucedanum japonicum* | [JF977817](https://www.ncbi.nlm.nih.gov/nuccore/JF977817) | *Peucedanum terebinthaceum* |
| [KP058321](https://www.ncbi.nlm.nih.gov/nuccore/KP058321) | *Peucedanum japonicum* | [JF977821](https://www.ncbi.nlm.nih.gov/nuccore/JF977821) | *Peucedanum terebinthaceum* |
| [KF806570](https://www.ncbi.nlm.nih.gov/nuccore/KF806570) | *Peucedanum japonicum* | [KF725034](https://www.ncbi.nlm.nih.gov/nuccore/KF725034) | *Peucedanum terebinthaceum* |
| [JF977805](https://www.ncbi.nlm.nih.gov/nuccore/JF977805) | *Peucedanum japonicum* | [KP682409](https://www.ncbi.nlm.nih.gov/nuccore/KP682409) | *Peucedanum terebinthaceum* |
| [DQ270200](https://www.ncbi.nlm.nih.gov/nuccore/DQ270200) | *Peucedanum ledebourielloides* | [EU236187](https://www.ncbi.nlm.nih.gov/nuccore/EU236187) | *Peucedanum turgeniifolium* |
| [JF977808](https://www.ncbi.nlm.nih.gov/nuccore/JF977808) | *Peucedanum ledebourielloides* | [KT282435](https://www.ncbi.nlm.nih.gov/nuccore/KT282435) | *Peucedanum urbanii* |
| [AM408876](https://www.ncbi.nlm.nih.gov/nuccore/AM408876) | *Peucedanum magalismontanum* | [KF806568](https://www.ncbi.nlm.nih.gov/nuccore/KF806568) | *Peucedanum wawrae* |
| [KF806573](https://www.ncbi.nlm.nih.gov/nuccore/KF806573) | *Peucedanum medicum* | [JF977824](https://www.ncbi.nlm.nih.gov/nuccore/JF977824) | *Peucedanum wawrae* |
| [JF977815](https://www.ncbi.nlm.nih.gov/nuccore/JF977815) | *Peucedanum medicum* | [JF977823](https://www.ncbi.nlm.nih.gov/nuccore/JF977823) | *Peucedanum wawrae* |
| [JF977812](https://www.ncbi.nlm.nih.gov/nuccore/JF977812) | Peucedanum medicum | [KF806569](https://www.ncbi.nlm.nih.gov/nuccore/KF806569) | *Peucedanum wulongense* |
| [JF977811](https://www.ncbi.nlm.nih.gov/nuccore/JF977811) | *Peucedanum medicum* | [AM408880](https://www.ncbi.nlm.nih.gov/nuccore/AM408880) | *Peucedanum zenkeri* |
| [AF077903](https://www.ncbi.nlm.nih.gov/nuccore/AF077903) | *Peucedanum morisonii* |  |  |
